# Ionophore-Based Ion-Selective Optodes Using Hydrocarbons as Ultralow-Polarity Media

**DOI:** 10.1021/acs.analchem.5c07237

**Published:** 2026-03-18

**Authors:** Aishwarya Patel, Krish Janmejay Patel, Simona Clement, Xuewei Wang

**Affiliations:** † Department of Chemistry, 6889 Virginia Commonwealth University, Richmond, Virginia 23284, United States; ‡ Department of Molecular and Cellular Biology, 8789 University of California, Davis, California 95616, United States

## Abstract

Ionophore-based ion-selective optodes, together with ion-selective electrodes, constitute a highly selective class of chemical sensors for detecting ionic species such as electrolytes. The choice of water-immiscible matrix in these sensors critically affects their analytical performance such as sensitivity and selectivity. In this study, we investigate ion-selective optodes that employ high–molecular-weight hydrocarbons as a new class of solvents. Compared with conventional plasticizers, hydrocarbons such as hexadecane dramatically enhance the sensitivity of ion-selective optodes by up to 4 orders of magnitude and substantially improve their selectivity. This enhancement arises from the stronger ionophore–ion binding affinity in the ultralow-polarity medium, leading to nearly complete extraction of the analyte ions and an exhaustive response with high sensitivity. The total color change is also expanded because the pH-sensitive chromoionophore exhibits a higher p*K*_a_ in hexadecane, ensuring its full protonation in the absence of the analyte ions. For sensing components that are insoluble in hydrocarbons, binary solvent mixtures consisting of a hydrocarbon and a plasticizer can be used while maintaining the enhanced response. This hydrocarbon-induced improvement in response is observed across a wide range of optodes incorporating various optical reporters (e.g., pH indicators and ionic dyes) and ionophores for different target ions (e.g., calcium and potassium ions). Two optode formats including liquid ion-selective optodes and paper-based ion-selective optodes are demonstrated using hexadecane as the medium. Collectively, these findings establish hydrocarbons as a new class of solvents that can significantly boost the analytical performance of ionophore-based ion sensors.

## Graphical abstract

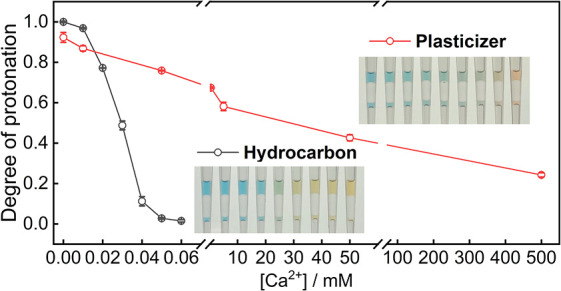

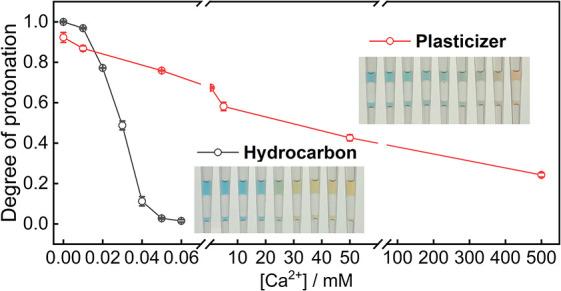

## Introduction

Ionophore-based sensors are among the most powerful technologies for detecting ionic species such as electrolytes. Ionophore-based ion-selective electrodes (ISEs) provide highly specific ion measurements in complex samples, including untreated whole blood, and have dominated the blood-electrolyte analyzer market for decades. Ionophore-based ion-selective optodes (ISOs) exhibit similar specificity and antifouling properties but do not rely on electrochemical cell configurations.^1–4^ Instead, ISOs employ a dye as an optical reporter to transduce ion–ionophore binding events. In both ISEs and ISOs, the ionophore is dissolved in a water-immiscible sensor phase that dictates selective ion binding. This water-immiscible matrix is integral to the function of these biphasic sensors. Ions exhibit different Gibbs free energies of partitioning depending on the matrix. The dielectric constant affects ion-pair formation and ion–ionophore binding. Matrices containing electronegative atoms may coordinate to cations in a nonspecific manner. These factors govern the selectivity, sensitivity, and response range of ionophore-based ion sensors. For ISOs, the role of the matrix is even more complex because the acid–base properties, spectrophotometric behavior, and dye solubility depend strongly on the medium. In addition, the viscosity of the medium influences membrane resistance for ISEs and response time for ISOs by altering diffusion coefficients.

Given the multifaceted importance of the water-immiscible matrix, a wide range of materials and solvents has been explored. The most classical matrix for both ISEs and ISOs is plasticized PVC. Common plasticizers include dioctyl sebacate (DOS) and 2-nitrophenyl octyl ether (NPOE), with dielectric constants of 4.2 and 21, respectively, representing some of the least and most polar solvents. Other ester-type plasticizers, such as sebacates, adipates, and phthalates, as well as chloroparaffins have also been employed.^5^ PVC may be replaced with other hydrophobic polymers such as polyurethane, polyacrylate, or silicone rubber.^6–8^ Incorporating PVC or other polymers generally increases the overall polarity of the matrix especially when the plasticizer is of low polarity.^9^ Self-plasticized matrices based on polysiloxanes, polyacrylates, and polymethacrylates have been developed to eliminate the use of plasticizers.^10–13^ More recently, lipid-based matrices such as aliphatic alcohols and triglycerides have been used for ISOs.^14,15^ Adsorption-based ISEs and ISOs have also been reported, in which the hydrophobic environment is provided by the sensing chemicals or coadsorbed hydrophobic molecules.^16–18^ Although the exact dielectric constants of many of these matrices are unspecified, their polarity is generally not lower than that of DOS. One exception is that some room-temperature-vulcanizing silicones can have a dielectric constant as low as 2.5–3.0.^10^ However, these moisture-triggered curing systems release polar byproducts such as alcohols and oximes.

The Bühlmann group reported ultralow-polarity matrices for ISEs based on fluorous membranes.^19,20^ Perfluorocarbons are among the least polar and least polarizable condensed phases. Their dramatically enhanced ion-pair formation constants, coupled with minimal coordinating ability toward interfering ions, enabled unprecedented selectivity in ISEs. However, relatively few sensors have been developed using fluorous membranes due to the need for synthesizing perfluorinated ionophores and ion exchangers. To the best of our knowledge, no ISOs using fluorous phases have been reported, likely because of the scarcity of perfluorinated dyes suitable as optical reporters. Surprisingly, another class of ultralow-polarity solvents, hydrocarbons, has been largely overlooked in ionophore-based sensors. Hydrocarbons possess dielectric constants comparable to perfluorocarbons and far lower than commonly used plasticizers. For example, the dielectric constant of octane (1.9–2.0) nearly matches that of perfluorooctane (1.8). Hydrocarbons also exhibit higher boiling points than fluorocarbons for *n* > 5, making them less volatile as sensor components.^21^ High-molecular-weight hydrocarbons (≥C14) have boiling points above 250 °C, comparable to plasticizers. In this work, we investigate hydrocarbons as solvents for ISOs and demonstrate that they dramatically enhance the sensitivity and selectivity of ISOs relative to plasticizers.

## Experimental Section

### Reagents

Buffer ingredients, salts, sensing chemicals (dyes, ion exchangers, and ionophores), and solvents were purchased from MilliporeSigma. Dyes include chromoionophore I (Ch-I, 9-(diethylamino)-5-(octadecanoylimino)-5*H*-benzo­[*a*]­phenoxazine), chromoionophore III (Ch-III, 9-(diethylamino)-5-[(2-octyldecyl)­imino]­benzo­[*a*]­phenoxazine), and methylene blue. Ionophores include potassium ionophore I (K–I, valinomycin), potassium ionophore II (K–II, bis­[(benzo-15-crown-5)-4′-ylmethyl] pimelate), potassium ionophore III (K–III, 2-dodecyl-2-methyl-1,3-propanediyl bis­[*N*-[5′-nitro­(benzo-15-crown-5)-4′-yl]­carbamate]), sodium ionophore IV (Na–IV, 2,3:11,12-didecalino-16-crown-5), sodium ionophore X (Na-X, 4-*tert*-butylcalix­[4]­arene-tetraacetic acid tetraethyl ester), calcium ionophore II (Ca–II, *N*,*N*,*N*′,*N*′-tetra­[cyclohexyl]­diglycolic acid diamide), calcium ionophore IV (Ca–IV, *N*,*N*-dicyclohexyl-*N*′,*N*′-dioctadecyl-3-oxapentanediamide), and calcium ionophore V (Ca–V, 10,19-Bis­[(octadecylcarbamoyl)­methoxyacetyl]-1,4,7,13,16-pentaoxa-10,19-diazacycloheneicosane). Ion exchangers include sodium tetrakis­[3,5-bis­(trifluoromethyl)­phenyl]­borate (NaTFPB) and potassium tetrakis­[3,5-bis­(trifluoromethyl)­phenyl]­borate (KTFPB). Solvents include dioctyl sebacate (DOS), dibutyl phthalate (DBP), hexadecane (HEX), and squalane.

### Preparation and Operation of Liquid ISOs

Sensing chemicals specified in the Results and Discussion Section are dissolved in the solvent using a sonication bath. The sensing oil and the aqueous solution are aspirated into 30 μL ClipTip pipet tips by an E1-ClipTip Electronic Multichannel Pipette. The pipet is programmed to perform repeated aspiration and dispensing steps for ∼15 min to move the liquids back and forth within the pipet tip to enable ion extraction and optical responses. Tests were conducted in triplicate.

### UV–Vis Absorption Spectroscopy

The absorption spectrum of the sensing oil is collected using a Varioskan LUX multimode microplate reader equipped with a μDrop plate that requires a liquid volume of 3–4 μL. The baseline of each spectrum is normalized so the absorbance value at 800 nm is zero, correcting for small instrument variations among different wells and tests.

### Preparation and Operation of Paper-Based ISOs

Sensing chemicals specified in the Results and Discussion section are dissolved in tetrahydrofuran. Two uL of the cocktail is pipetted onto Whatman filter paper (Grade 5) to create an ISO. The modified paper is dried under vacuum for 1 h and circular sensors with a diameter of 1″ are cut from the paper using a hole punch. The sensor is pressed onto parafilm and 50 μL of the aqueous solution is added onto the sensor. Photographs are taken using an iPhone 13 after 5 min of incubation. The hue of each optode is analyzed using Adobe Capture. Tests were conducted in triplicate.

## Results and Discussion

### Solubility of Sensing Chemicals in Hexadecane

Perfluorocarbons have ultralow polarizability due to the high electronegativity of fluorine, which prevents them from forming instantaneous dipole moments capable of interacting with nonperfluorinated compounds. As a result, they are extremely hydrophobic and lipophobic. Commercially available sensing chemicals used in ion sensors, such as ionophores, ion exchangers, and dyes, usually cannot be dissolved in perfluorocarbons. In contrast, hydrocarbons may dissolve these sensing chemicals, although their solvation capability is generally lower than that of more polar plasticizers which can form stronger intermolecular interactions such as hydrogen bonds and dipole–dipole forces. Hexadecane (HEX) is chosen as the representative hydrocarbon medium for liquid ISOs because it is a nonvolatile liquid at room temperature, with a melting point of 18 °C and a boiling point of 287 °C. HEX also has a viscosity of only 3 mPa·s, comparable to or even lower than many plasticizers and branched hydrocarbons such as squalane. This relatively low viscosity increases the diffusion coefficients of sensing chemicals and thereby accelerates the optical response. If the sensor is intended for low-temperature use, shorter hydrocarbons such as tetradecane can be employed to prevent solidification of the oil phase.

Table 1 summarizes the solubility, chemical structure, dipole moment, and logP of a range of ionophores and chromoionophores commonly used to prepare ISOs. Ionophores with large dipole moments show very low solubility in HEX (<5 μM), as seen for Ca–VI, K–I, K–II, and K–III. In contrast, Ca–IV, Na-X, Na–IV, and Ca–II with much lower dipole moments (1–4 D) display substantially higher solubility (in or near the millimolar range), allowing them to be used to formulate ISOs. Both Ch-I and Ch-III, which also have low dipole moments, exhibit solubilities greater than 0.25 mM. A comparison of Ca–IV and Ca–II suggests that unbranched long alkyl chains increase solubility. This is expected because long, flexible alkyl chains resemble the hydrocarbon solvent, enabling enhanced London dispersion interactions. However, long alkyl chains alone do not guarantee high solubility when the rest of the molecule is highly polar, as seen for Ca–V and K–III. Compared with dipole moment, calculated logP correlates less closely with measured solubility in HEX. Notably, these dipole moments are gas-phase values calculated in Avogadro for a single optimized conformation of each molecule and do not account for conformational averaging. They are used here only as a qualitative descriptor of polarity within this limited series of compounds. We also tested the Hansen Solubility Parameters in Practice (HSPiP) software, which estimates solubility by quantifying dispersion, polar, and hydrogen-bonding interactions. However, the software was unable to handle these large solutes and did not produce reasonable solubility estimates.

**Table 1:**
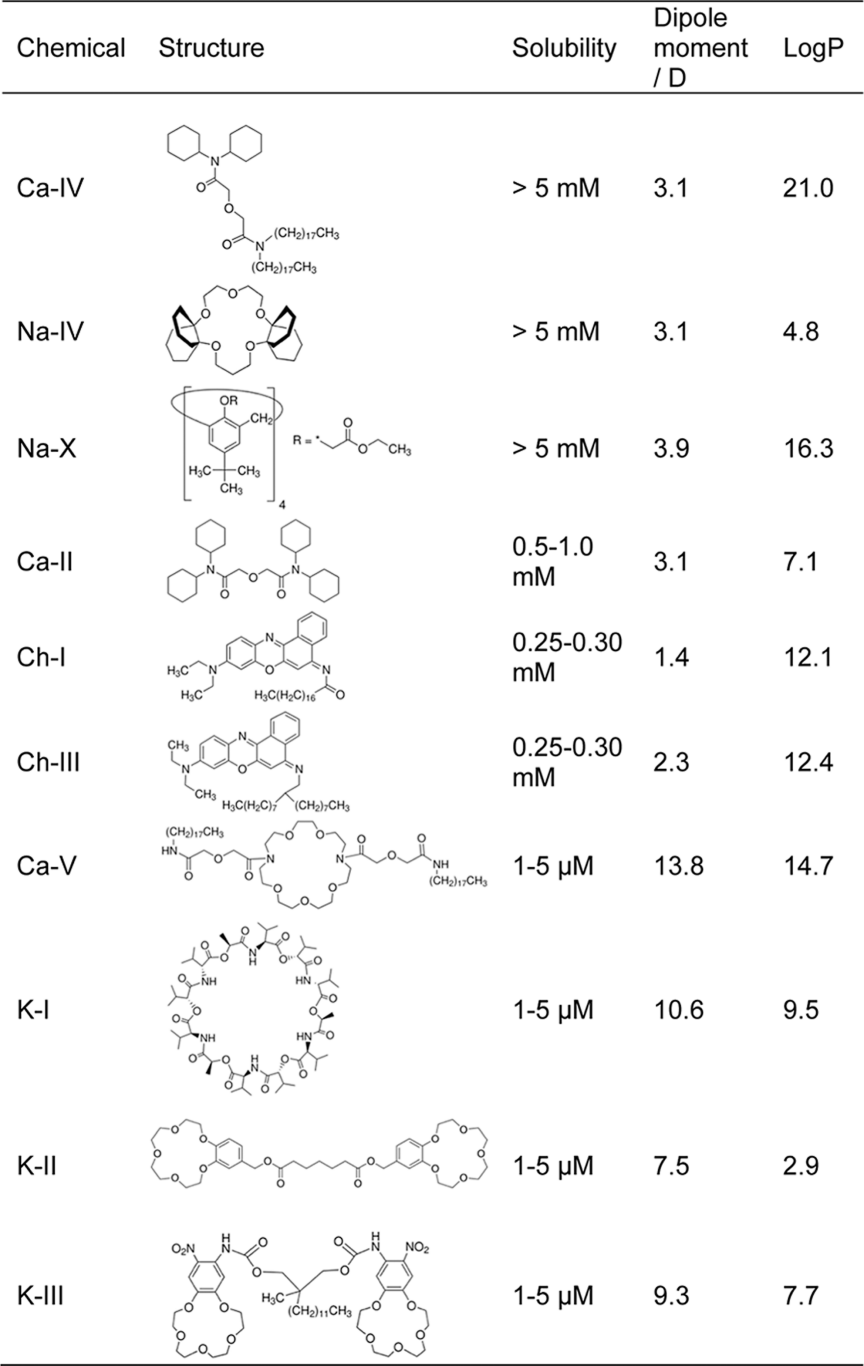
Solubility of Ionophores and Chromoionophores in HEX^a^

a Molecular dipole moments are calculated using Avogadro 1.2 after the molecular geometries are optimized using the “Optimize Geometry” function. LogP values are calculated by ChemDraw 22.0.

Although chromoionophores and some neutral ionophores can be dissolved in HEX, ion exchangers such as NaTFPB and KTFPB are salts with small inorganic cations and show poor solubility (<5 μM). Because ion exchangers are essential components for cation-selective optodes, it initially appears that functional ISOs cannot be formulated in HEX. However, the solubility of these ion exchangers dramatically increases when mixed with more soluble sensing components. For example, when 100 μM NaTFPB is added to HEX in the presence of 300 μM Ca–IV, both chemicals are fully soluble, enabling preparation of a functional ISO. Likewise, in the presence of 100 μM Ch-III, 100 μM NaTFPB also becomes fully soluble. Ionophores typically bind both analyte and interference ions, although with much higher affinity for the analyte.^22^ In the presence of Ca–IV, Na^+^ can be partially complexed, increasing its solubility in HEX. Chromoionophores also contain electronegative amine groups capable of binding Na^+^, enhancing its solvation in HEX. As a result, it is feasible to formulate fully functional ISOs using HEX as the sole solvent.

### Liquid ISOs Using Hexadecane as the Solvent

Our group has been developing liquid-state ISOs based on water-immiscible plasticizers.^23–25^ Unlike nanoparticle- or microparticle-based ISOs, which rely on surfactants that potentially compromise selectivity, liquid-state ISOs do not require surfactants. Unlike traditional polymeric-membrane ISOs, liquid ISOs do not incorporate polymers such as PVC, which can impart fixed charges and alter membrane polarity. This makes liquid ISOs a simple and powerful platform for systematically evaluating different solvents. We have developed two configurations of liquid ISOs: (1) pressure-driven droplet microfluidics in microchannels^23,24^ and (2) stepper-motor–driven push–pull fluidics in millichannels.^25^ In the push–pull platform, an electronic pipet executes programmed aspiration and dispensing cycles to mix an oil-based ISO with an aqueous sample inside a pipet tip, which will be used in this work to demonstrate ISOs of various formulations.

We formulated liquid ISOs by dissolving the same concentrations of Ch-III, NaTFPB, and Ca–IV in either HEX or DOS. As shown in Figure 1, using HEX as the ISO medium dramatically enhances optode sensitivity. Because both Ch-III and NaTFPB are present at 100 μM in the oil phase, the theoretical exhaustive sensing mode predicts a dynamic range of 0–50 μM for Ca^2+^ in the aqueous phase.^25–27^ For the HEX-based ISO, the degree of protonation of Ch-III decreases from approximately 100% to only 2.8% as the aqueous Ca^2+^ concentration increases from 0 to 50 μM, consistent with exhaustive-response behavior and indicating high extraction efficiency of Ca^2+^ into the sensing oil. In contrast, when DOS is used as the solvent (Figure 1C), the sensitivity is much lower. The degree of protonation remains 24.3% even at an aqueous Ca^2+^ concentration of 500 mM, indicating a dramatically reduced Ca^2+^ extraction efficiency. Overall, the response of the DOS-based ISO spans multiple orders of magnitude and is better described by the classical ion-exchange equilibrium theory, whereas the HEX-based ISO exhibits a much narrower dynamic range that matches the exhaustive-response model (Figure S1).

**Figure 1:**
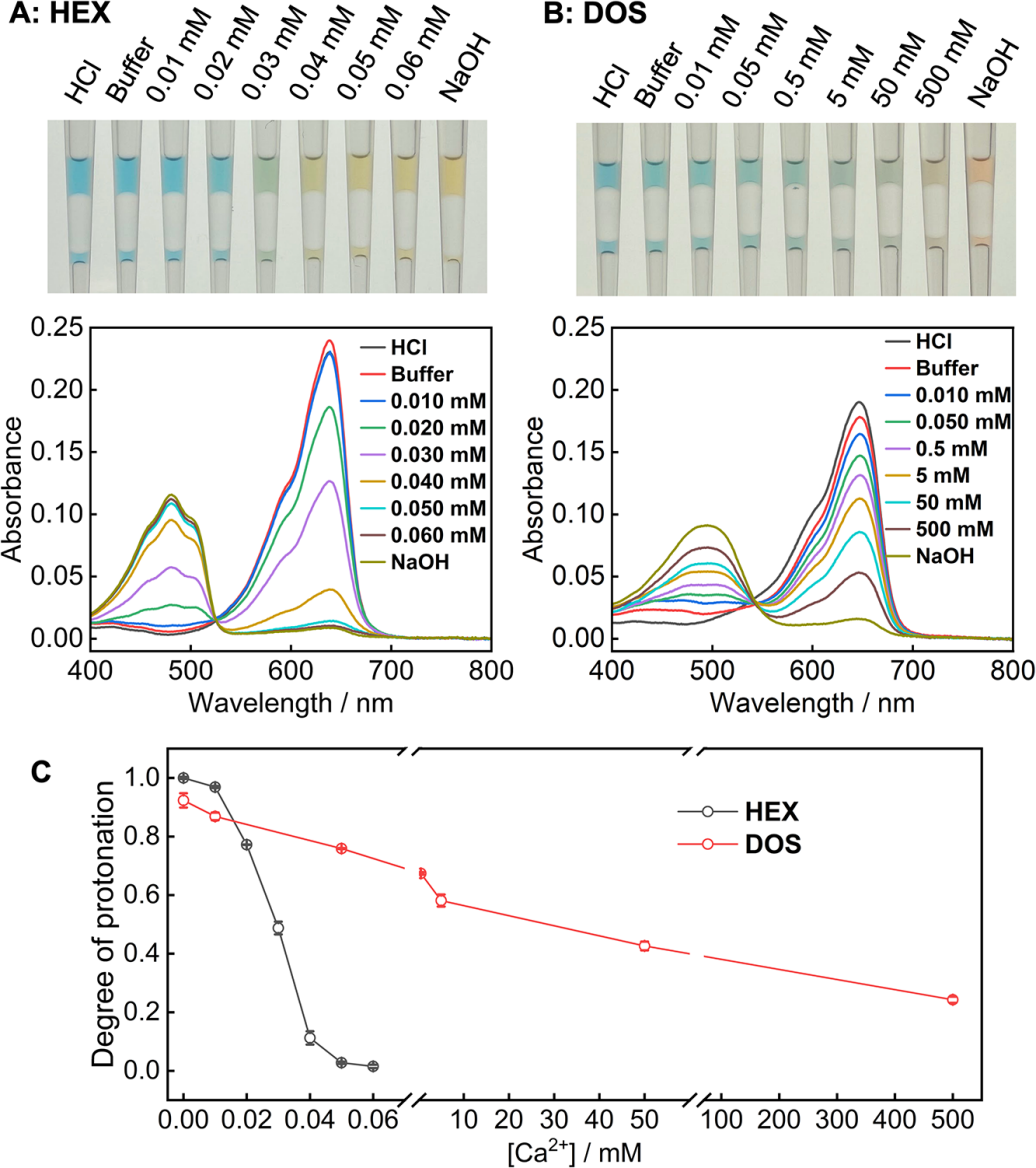
Photographs and absorption spectra of liquid ISOs formulated with HEX (A) or DOS (B) after equilibration with 0.1 M HCl, 0.1 M NaOH, 0.1 M HEPES-Tris buffer at pH 7.4, and the buffer containing different concentrations of CaCl_2_. Both sensing oils contain 100 μM Ch-III, 100 μM NaTFPB, and 300 μM Ca–IV. The volume of each phase is 8 μL. The degree of protonation of Ch-III is calculated based on absorbance at ∼639 nm for HEX and ∼647 nm for DOS and plotted against the concentration of Ca^2+^ in the buffer (C). The absorbance of the HEX-based oil is slightly lower for HCl than for buffer, which is because the dye slightly leaks into the aqueous phase at pH 1.0. In this case, the absorbance for buffer is used to represent fully protonated Ch-III for calculating the degree of protonation.

The sensitivity of a cation ISO employing a pH indicator as the reporter is governed by the ion-exchange equilibria between analyte ions and protons. A lower basicity (lower p*K*_a_) of the chromoionophore favors response toward analyte cations because the Gibbs free energy of deprotonation is lower. However, when we measured the pH response of Ch-III in HEX and DOS, we found that the apparent p*K*_a_ of Ch-III is actually much higher in HEX (Figure S2). This would make Ca^2+^ extraction more difficult and therefore cannot account for the enhanced response observed in HEX. The most plausible explanation is a significantly increased ionophore–Ca^2+^ binding affinity in the ultralow-polarity solvent. In general, cation–dipole interactions are known to increase as the polarity of the solvent decreases.^28–31^ Hydrocarbons, unlike plasticizers, cannot effectively solvate cations due to the absence of electronegative atoms, making cations more available for complexation by the ionophore. Moreover, low-polarity solvents cannot perturb the lone-pair electrons on donor atoms in the ionophore, further favoring ion coordination.^32^ Similar ultralow-polarity hydrocarbons such as squalane also yield fully exhaustive responses with high sensitivity (Figure S3). Other factors such as enhanced ion pairing interaction and altered ionophore–ion binding stoichiometry may also play a role.

One could argue that the same exhaustive response might be achieved by simply increasing the ionophore concentration in traditional plasticizers such as DOS. However, as shown in Figure 2, even when the molar ratio of ionophore to ion exchanger in DOS is increased to 10:1, the colorimetric response remains dramatically smaller than that of the HEX-based ISO using only a 3:1 ratio. Worse still, the degree of protonation of Ch-III in the absence of Ca^2+^ decreases significantly as the concentration of Ca–IV increases, narrowing the useable optical signal window for Ca^2+^. At an ionophore-to-ion-exchanger ratio of 60:1, Ch-III becomes nearly fully deprotonated even in the absence of Ca^2+^. Thus, the performance of DOS-based ISOs, regardless of ionophore loading, is substantially inferior to that of HEX-based ISOs. The deprotonation of Ch-III in DOS in the absence of Ca^2+^ is most likely due to binding of Ca–IV to Na^+^ introduced by the ion exchanger.^22^ As the concentration of Ca–IV increases, the exchange of Na^+^ in the oil phase by protons in the buffer becomes increasingly difficult because Ca–IV withholds Na^+^ tightly. In contrast, large excesses of Ca–IV are unnecessary when HEX is used. Although HEX dramatically boosts the response toward Ca^2+^, its response toward Na^+^ increases only slightly relative to DOS (Figure S4), resulting in substantially improved selectivity. The sensitivity to Ca^2+^ is more than 5000-fold higher than to other cations such as Na^+^, K^+^, and Mg^2+^.

**Figure 2:**
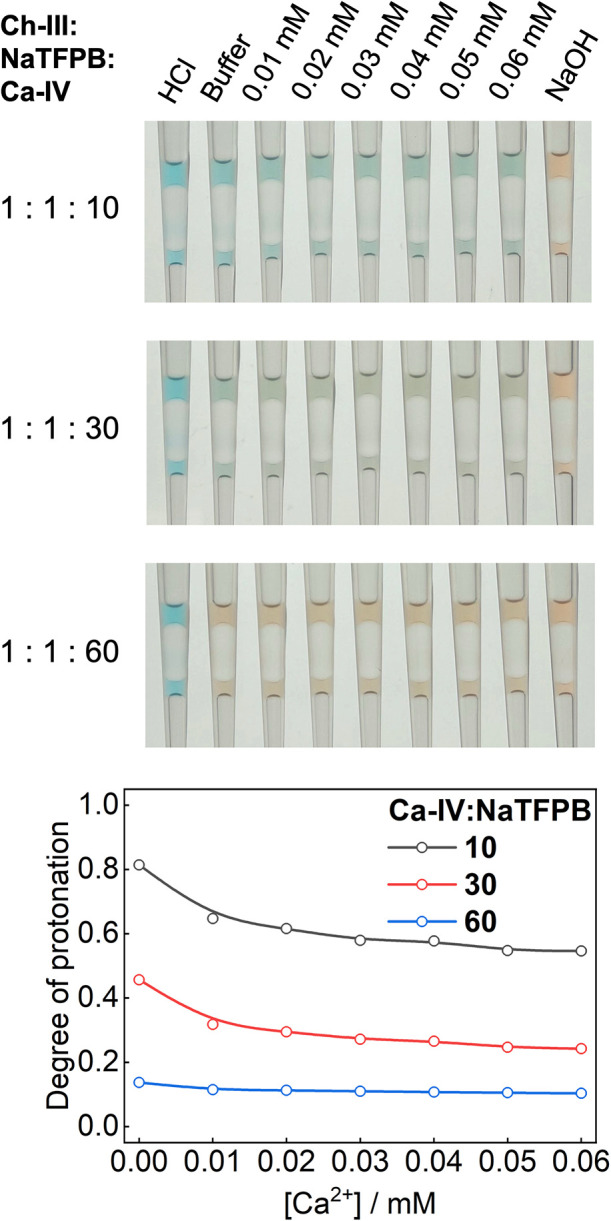
Response of DOS-based Ca^2+^-ISOs containing 100 μM Ch-III, 100 μM NaTFPB, and different concentrations of Ca–IV (1 mM, 3 mM, or 6 mM). Experimental conditions are the same as those for Figure 1.

We also formulated HEX-based liquid ISOs using Ch-I, a less basic chromoionophore. Again, NaTFPB becomes soluble in HEX in the presence of more soluble components including Ca–IV and Ch-I. As shown in Figure S5, the HEX-based ISO is again dramatically more sensitive than the DOS-based ISO. The HEX-based response is fully saturated at 70 μM Ca^2+^, matching the 150 μM NaTFPB content, whereas the DOS-based ISO remains unsaturated even at 50 mM Ca^2+^.

### Liquid ISOs Using Binary Solvent Mixtures of Hexadecane and Plasticizer

Many sensing chemicals remain only sparingly soluble in HEX, even in the presence of more soluble components. For example, permanently cationic dyes such as methylene blue, rhodamine 6G, and Thioflavin T can be used as pH-independent optical reporters in ISOs.^24,33–35^ They are only sparingly soluble in HEX, even when mixed with highly soluble ionophores. Consequently, direct formulation of ISOs in pure HEX using these ionic dyes is challenging. To overcome this limitation, we first prepared a DOS-based sensing oil containing the ionic dye, NaTFPB, and Ca–IV, and then mixed HEX into this sensing oil. As shown in Figure 3, when the sensing oil initially formulated in DOS is blended with HEX, the resulting ISO exhibits substantially higher sensitivity than when the same sensing oil is mixed with DOS alone or with a more polar plasticizer such as DBP. Although the improvement is less dramatic than the multiorder-of-magnitude enhancement observed for fully HEX-soluble formulations, the inclusion of HEX in binary solvent mixtures nevertheless provides a new and effective approach to boosting the response of ISOs that incorporate sensing chemicals insoluble in pure HEX. This represents another important distinction from fluorous media, since fluorous solvents are incompatible with plasticizers and may not be blended with them.

**Figure 3:**
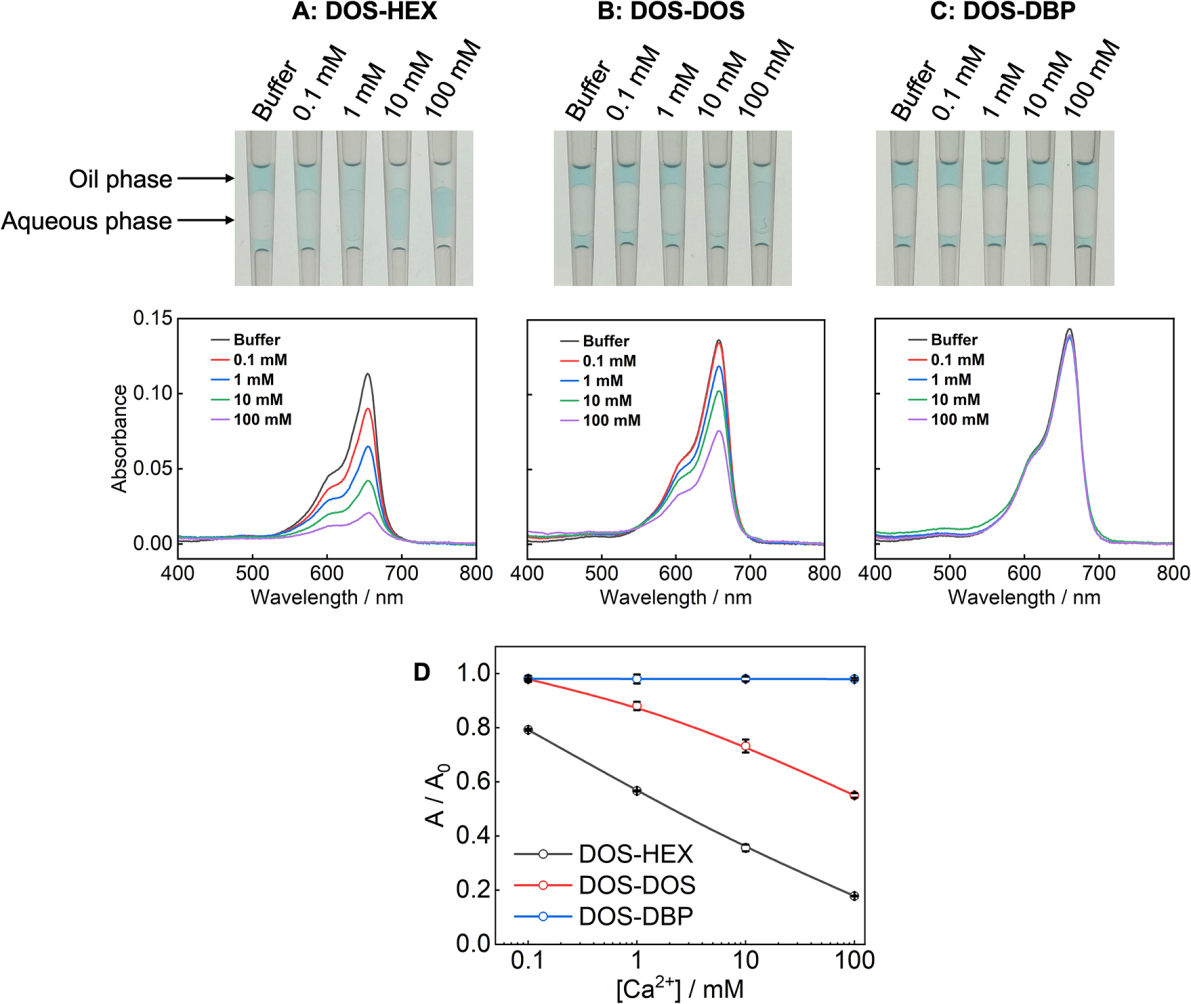
Photographs and absorption spectra of Ca^2+^ ISOs prepared by mixing DOS-based sensing oil with HEX (A), DOS (B), or DBP (C) at a 1:1 volume ratio. The maximum absorbance in each spectrum is used to plot response curves (D), in which “A” denotes the absorbance of each oil phase and “A_0_” denotes the absorbance of the oil phase after equilibration with the Ca^2+^-free buffer. To prepare the DOS-based sensing oil, DOS containing 100 μM NaTFPB and 300 μM Ca–IV is mixed with an aqueous solution containing 1 mM chloride salt of methylene blue. Two solutions are vortexed in a centrifuge tube so methylene blue cations are extracted into the oil to expel Na^+^ into the aqueous phase. Then the oil phase is carefully collected after centrifugation and used as the sensing oil to be mixed with the ancillary solvent.

When the ionophore is insoluble in HEX, a binary solvent mixture can also serve as an effective way to enhance the ISO response. Figure 4 shows the response of a DOS-based sensing oil mixed with HEX, DOS, or DBP for K^+^ sensing. Although K–III is sparingly soluble in pure HEX, it is highly soluble in DOS. Again, as the polarity of the solvent mixture decreases with the inclusion of HEX, the sensitivity of the ISO increases. Together, these results indicate that ultralow-polarity hydrocarbons can be used as ancillary solvents to enhance the sensitivity of a wide range of ISOs formulated from diverse sensing chemicals.

**Figure 4:**
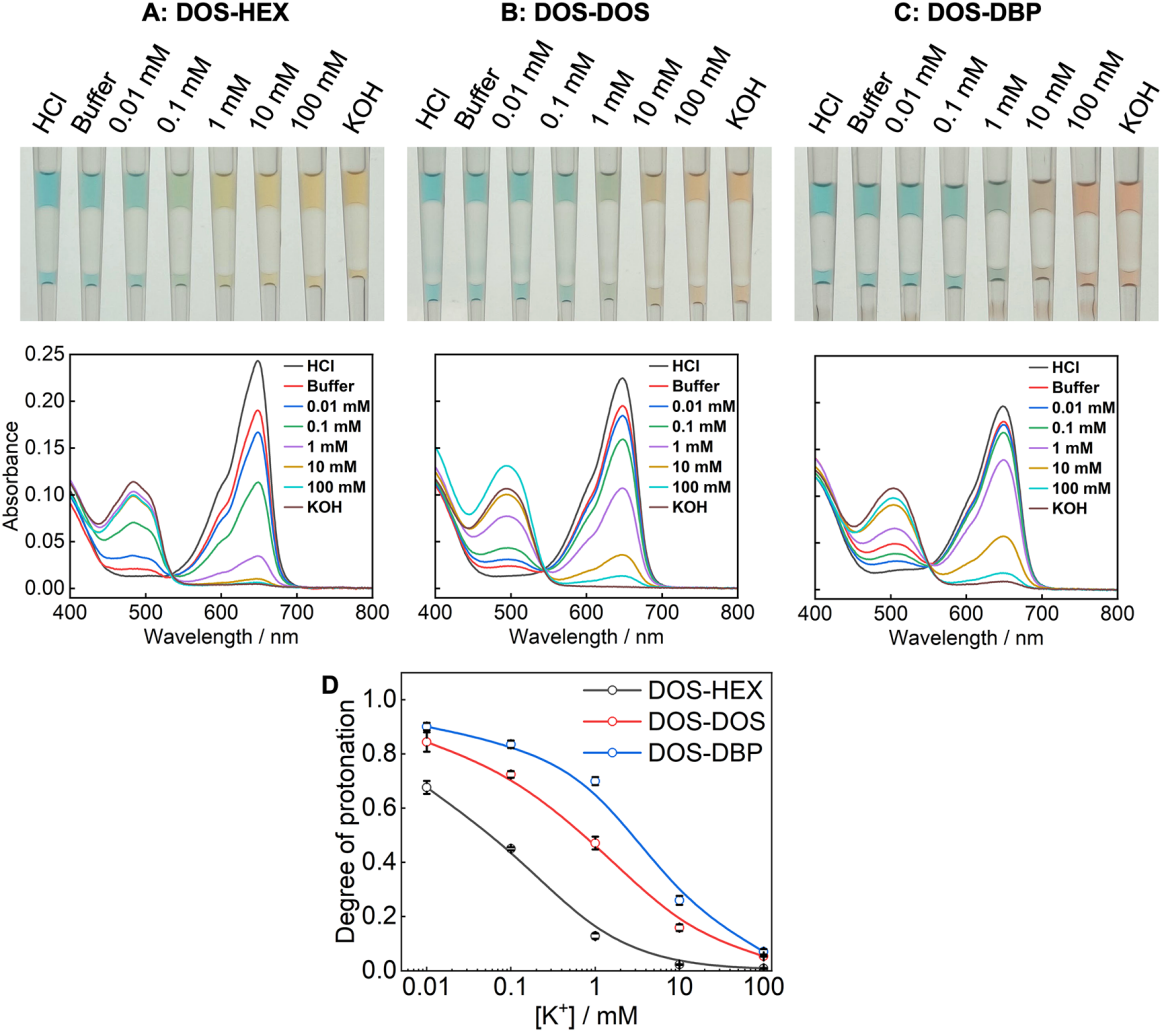
Photographs and absorption spectra of K^+^ ISOs prepared by mixing DOS-based sensing oil with HEX (A), DOS (B), or DBP (C) at a 1:4 volume ratio. The maximum absorbance in each spectrum is used to plot response curves (D). The DOS-based sensing oil contains 250 μM Ch-III, 250 μM NaTFPB, and 750 μM K–III before mixing with an ancillary solvent. The aqueous phase is 0.1 M HCl, 0.1 M KOH, 0.01 M HEPES-Tris buffer at pH 7.4, and the buffer containing different concentrations of KCl.

### Hexadecane for Paper-Based ISOs

The liquid ISOs are suited for fluidics-based sensing platforms such as push–pull millifluidics and droplet microfluidics. Planar ISOs, including paper-based formats, represent a different class of ion sensors that offer simple operation without the need for motors.^3^ In our recent work, we fabricated paper-based ISOs using Ch-III, NaTFPB, and Ca–II in the presence of plasticizers such as DOS and NPOE.^36^ Even when employing the most basic chromoionophore commercially available, the optode appears far from full protonation even in an acidic buffer without analyte ions, suggesting that protons from the aqueous phase cannot effectively displace Na^+^ in the ISO. Consequently, these sensors fail to function at physiological pH 7.4. Because HEX increases the apparent p*K*_a_ of Ch-III, we investigated whether it could overcome this limitation. As shown in Figure 5, the DOS-based ISO on paper indeed exhibits negligible response because Ch-III remains deprotonated regardless the concentration of Ca^2+^. In contrast, replacing DOS with HEX renders the optode bluish at pH 7.4, enabling Ca^2+^ detection via Ca^2+^-induced deprotonation of Ch-III. Although paper-based ISOs are more complex than liquid ISOs owing to the use of tetrahydrofuran during fabrication and interactions between sensing components and cellulose fibers, these results illustrate that ultralow-polarity hydrocarbons offer a promising approach for modulating the analytical performance of solid-state ISOs as well.

**Figure 5:**
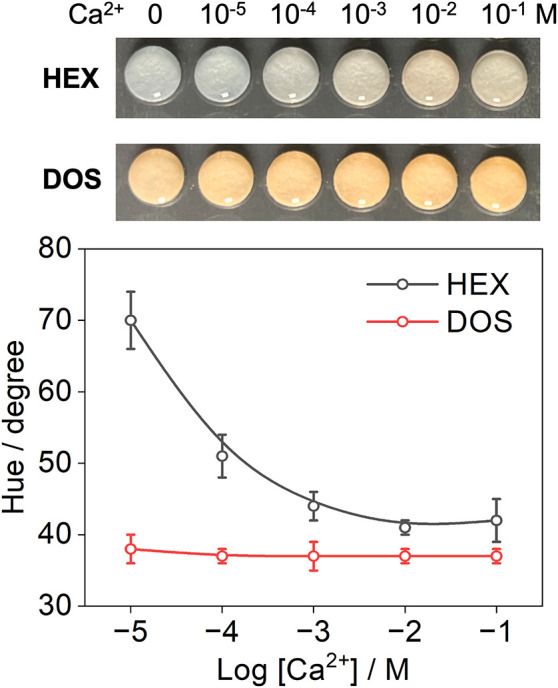
Colorimetric response of the paper-based ISOs using HEX or DOS. The aqueous solution is HEPES-Tris buffer at pH 7.4 containing different concentrations of Ca^2+^. The cocktail to prepare the ISO is tetrahydrofuran containing 1.0 mmol/L Ch-III, 1.2 mmol/L NaTFPB, 3.5 mmol/L Ca–II, and 10 mg/mL HEX or DOS.

## Conclusion

Hydrocarbons such as HEX enhance the response of liquid ISOs when used either as the sole solvent or as a component in a binary solvent mixture. Compared to previously reported ultralow-polarity sensor matrices based on perfluorinated solvents and polymers, the hydrocarbon medium is compatible with commercially available sensing components for ionophore-based sensors. In future work, custom synthesis of sensing chemicals with improved solubility in hydrocarbons may further expand the range of analytes that can be detected. Incorporating hydrocarbons into sensor matrices may also benefit other ISO formats, such as membranes and nano/microparticles, by modulating the ionophore binding affinity and the p*K*_a_ of optical reporters. Moreover, given the previously demonstrated advantages of fluorous membrane–based ISEs, the potential role of hydrocarbons in enhancing the performance of ISEs also warrants investigation.

## Supplementary Material



## Notes

Figures showing fitting of the optical responses using theoretical models, the pH sensitivity of Ch-III in different solvents, the selectivity of the Ca^2+^ ISOs based on HEX and DOS, the response of Ca^2+^ ISOs using squalane as the solvent, and the response of Ca^2+^ ISOs using Ch-I as the optical reporter (PDF)
