# Supplementary material for: Ionophore-Based Ion-Selective Optodes Using Hydrocarbons as Ultralow-Polarity Media
Source: Anal Chem. 2026 Mar 18;98(12):9038–45. doi: 10.1021/acs.analchem.5c07237 (PMC13044874; doi:10.1021/acs.analchem.5c07237)
Supplement: Supplementary file 1 [file ac5c07237_si_001.pdf]

Supporting Information

Ionophore-Based Ion-Selective Optodes Using Hydrocarbons as Ultralow-Polarity Media

Aishwarya Patel[1 #], Krish Janmejay Patel[2 #], Simona Clement[1], Xuewei Wang[1 *]

[1] Department of Chemistry, Virginia Commonwealth University, Richmond, VA 23284, United States

[2] Department of Molecular and Cellular Biology, University of California, Davis, CA 95616, United States

Corresponding author: Xuewei Wang

*Email: wangx11@vcu.edu

[#]: These authors contributed equally to this work

Table of Contents

Figure S1. Fitting of the responses of hexadecane (HEX)- and dioctyl sebacate (DOS)-based ISOs using the equilibrium and exhaustive response theories. *S2*

Figure S2. Response of Ch-III in HEX and DOS toward pH of the aqueous phase.......*S3*

Figure S3. Response of liquid ISOs prepared in squalane. *S3*

Figure S4. Selectivity of the HEX and DOS-based Ca^{2+} ISOs. *S4*

Figure S5. Photographs and absorption spectra of liquid ISOs based on HEX (A) or DOS (B) *S4*

References...*S5*

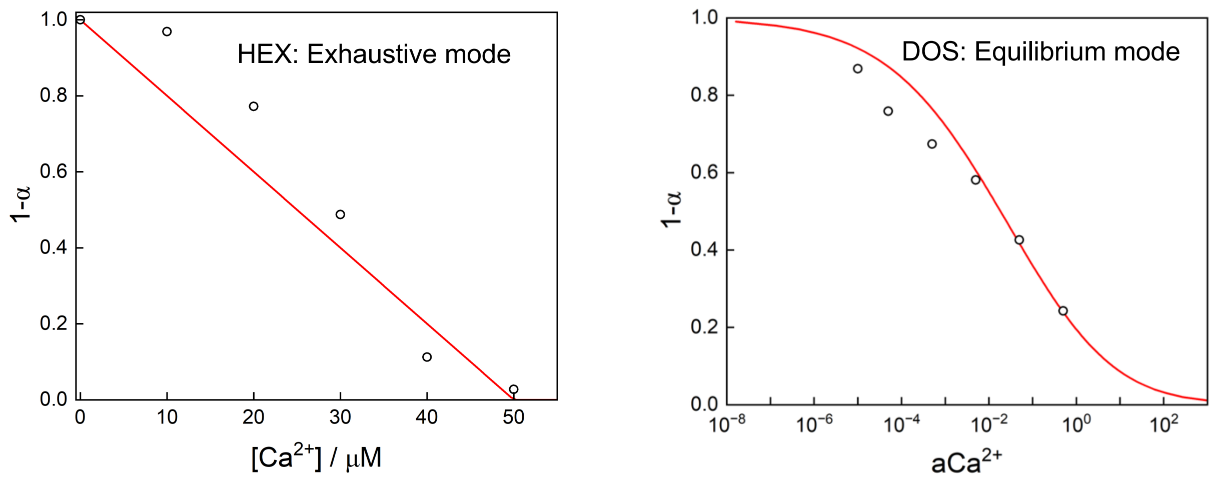

Figure S1. Fitting of the responses of hexadecane (HEX)- and dioctyl sebacate (DOS)-based ISOs using the equilibrium and exhaustive response theories. MATLAB is used for the equilibrium sensing mode. The experimental data are those shown in Figure 1C. In an ideal exhaustive sensing mode, any Ca^{2+} in the aqueous phase is extracted into the optode. Because Ch-III and NaTFPB are each present at 100 μM in the oil phase, the extraction of each Ca^{2+} ion results in the deprotonation of two Ch-III molecules. In the absence of Ca^{2+} in the buffer, Ch-III is fully protonated. Thus, the degree of protonation (1 − α) is inversely proportional to the molar concentration of Ca^{2+} in the aqueous phase, $C_{Ca}(aq)$ (M):

$$1-\alpha=\frac{n_{HInd}}{n_{Ind(tot)}}=\frac{n_{Ind}(tot)-2\times C_{Ca}(aq)\times V_{aq}}{n_{Ind(tot)}}=1-2\times10^{4}\times C_{Ca}(aq).$$

Here, n_{HInd} is the number of moles of protonated Ch-III, $n_{Ind(tot)}$ is the total number of moles of Ch-III (0.8 nmol in 8 μL of oil at 100 μM), and V_{aq} is the volume of the aqueous phase, which is equal to the volume of the oil phase. For the same oil formulation, the classical equilibrium response theory (equation 15 in reference 3)[3] predicts a reverse S-curve as a function of the logarithm of the Ca^{2+} activity over a much broader range. Despite some deviations, the response of the HEX-based ISO generally follows the exhaustive response mode, whereas the DOS-based ISO matches the equilibrium response mode, confirming that HEX provides a dramatically higher Ca^{2+} extraction efficiency. The deviation between the experimental data and the fits arises from several factors. For the HEX-based ISO, the high pK_a of Ch-III, nonspecific weak binding of Na^+ to the ionophore, and the finite binding constant of the ionophore for Ca^{2+} cause departures from the ideal linear exhaustive response. For the DOS-based ISO, a significant fraction of Ca^{2+} is extracted into the oil phase at low aqueous Ca^{2+} concentrations, given the equal phase volumes. This violates the equilibrium sensing assumption of negligible depletion of the primary ion in the aqueous phase and results in larger responses.

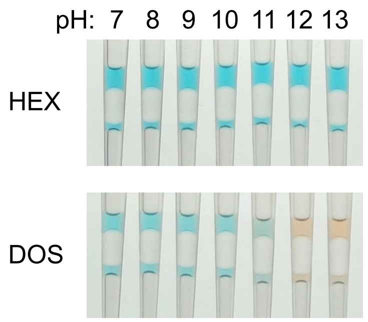

Figure S2. Response of Ch-III in HEX and DOS toward pH of the aqueous phase. In addition to 100 μM Ch-III, 100 μM NaTFPB is also dissolved in the solvent to enable extraction of protons into the oil phase via ion exchange. The universal buffer (Britton–Robinson buffer) is used for pH 7-12 and 0.1 M NaOH is used for pH 13.

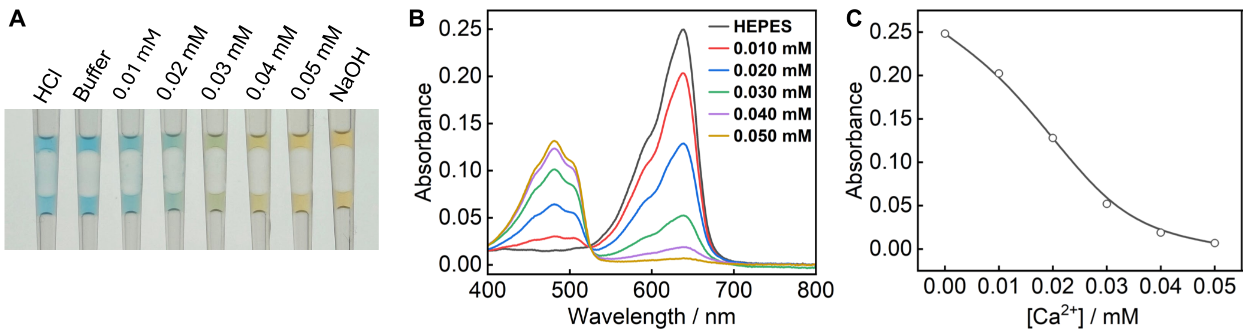

Figure S3. Colorimetric response of liquid ISOs prepared in squalane (A: photograph; B: absorption spectra; C: absorbance-based calibration curve). The concentration of Ch-III, NaTFPB, and calcium ionophore IV (Ca-IV) is 100 μM, 100 μM, and 300 μM, respectively. The buffer is 0.1 M HEPES-Tris buffer at pH 7.4. The concentration of HCl and NaOH is 0.1 M.

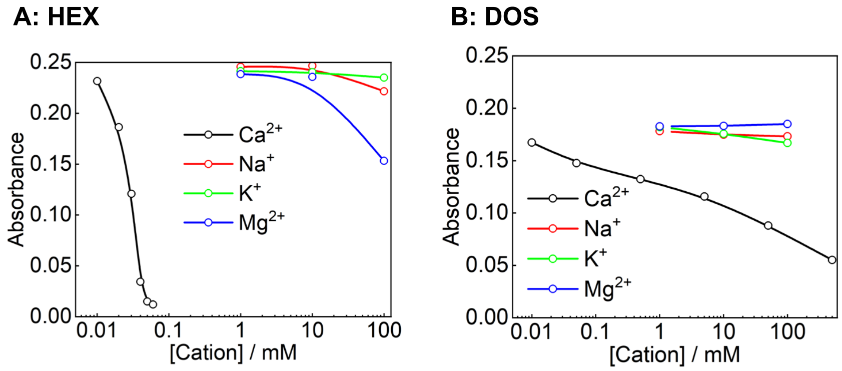

Figure S4. Selectivity of the HEX (A) and DOS (B)-based Ca^{2+} ISOs. The oil formulations and experimental conditions are the same as those for Figure 1. The buffer is 0.1 M HEPES-Tris buffer at pH 7.4.

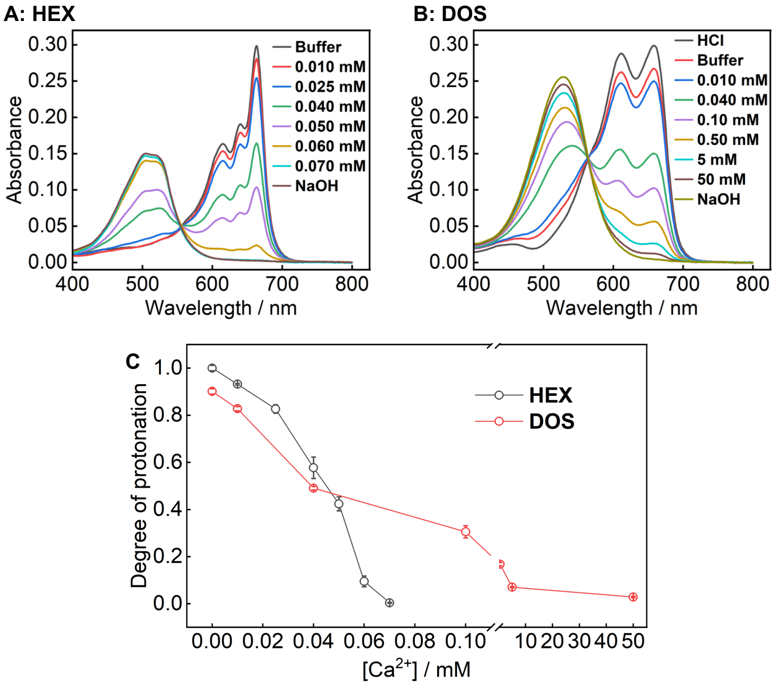

Figure S5. Photographs and absorption spectra of liquid ISOs based on HEX (A) or DOS (B) after equilibration with 0.1 M HCl, 0.1 M NaOH, 0.1 M HEPES-Tris buffer at pH 7.4, and the buffer containing different concentrations of $CaCl_2$. Both sensing oils contain 100 μM Ch-I, 150 μM NaTFPB, and 800 μM Ca-IV. The volume of each phase is 8 μL. The degree of protonation of Ch-I is calculated based on absorbance at 663 nm for HEX and 659 nm for DOS and plotted against the concentration of Ca^{2+} in the buffer (C). The absorbance of HEX-based oil after equilibration with buffer is used as the absorbance of the fully protonated Ch-I because Ch-I partially leaks to the aqueous phase when it is 0.1 M HCl.

References

1. Xie, X.; Zhai, J.; Bakker, E. PH Independent Nano-Optode sensors based on exhaustive Ion-Selective nanospheres. *Analytical Chemistry* **2014**, *86* (6), 2853–2856.
2. Xie, X.; Zhai, J.; Crespo, G. A.; Bakker, E. Ionophore-Based Ion-Selective Optical NanoSensors operating in exhaustive sensing mode. *Analytical Chemistry* **2014**, *86* (17), 8770–8775.
3. Mistlberger, G.; Crespo, G. A.; Bakker, E. Ionophore-Based optical sensors. *Annual Review of Analytical Chemistry* **2014**, *7* (1), 483–512.
